# Recent Applications of Carbon Nanotubes in Organic Solar Cells

**DOI:** 10.3389/fchem.2021.733552

**Published:** 2022-01-06

**Authors:** Edigar Muchuweni, Edwin T. Mombeshora, Bice S. Martincigh, Vincent O. Nyamori

**Affiliations:** School of Chemistry and Physics, University of KwaZulu-Natal, Durban, South Africa

**Keywords:** carbon nanotubes, organic solar cells, photoactive layer, hole transport layer, electron transport layer

## Abstract

In recent years, carbon-based materials, particularly carbon nanotubes (CNTs), have gained intensive research attention in the fabrication of organic solar cells (OSCs) due to their outstanding physicochemical properties, low-cost, environmental friendliness and the natural abundance of carbon. In this regard, the low sheet resistance and high optical transmittance of CNTs enables their application as alternative anodes to the widely used indium tin oxide (ITO), which is toxic, expensive and scarce. Also, the synergy between the large specific surface area and high electrical conductivity of CNTs provides both large donor-acceptor interfaces and conductive interpenetrating networks for exciton dissociation and charge carrier transport. Furthermore, the facile tunability of the energy levels of CNTs provides proper energy level alignment between the active layer and electrodes for effective extraction and transportation of charge carriers. In addition, the hydrophobic nature and high thermal conductivity of CNTs enables them to form protective layers that improve the moisture and thermal stability of OSCs, thereby prolonging the devices’ lifetime. Recently, the introduction of CNTs into OSCs produced a substantial increase in efficiency from ∼0.68 to above 14.00%. Thus, further optimization of the optoelectronic properties of CNTs can conceivably help OSCs to compete with silicon solar cells that have been commercialized. Therefore, this study presents the recent breakthroughs in efficiency and stability of OSCs, achieved mainly over 2018–2021 by incorporating CNTs into electrodes, active layers and charge transport layers. The challenges, advantages and recommendations for the fabrication of low-cost, highly efficient and sustainable next-generation OSCs are also discussed, to open up avenues for commercialization.

## Introduction

Recently, there has been a dramatic increase in the global demand for renewable and green energy sources due to the exhaustion and environmental issues associated with conventional energy sources, such as fossil fuels and nuclear energy (Rego de Vasconcelos and Lavoie, 2019; Ashok et al., 2020; Lin et al., 2020; Subhan et al., 2020; Tiwari et al., 2020). In this regard, solar energy, a low-cost, renewable, naturally abundant and clean energy source, has attracted enormous research effort as a promising alternative to traditional energy sources (Subramanyam et al., 2019; Zhong et al., 2020; Rabaia et al., 2021). Among the new-generation photovoltaic devices that convert solar energy into electricity, organic solar cells (OSCs) are being widely investigated owing to their low production cost, facile fabrication procedure, abundance of raw materials, easy scalability, lightweight, excellent flexibility and environmentally friendly nature (Gusain et al., 2019; Lee, 2019; Li et al., 2020a; Shah et al., 2020; Shoyiga et al., 2020; Liu et al., 2021).

**FIGURE 1:**
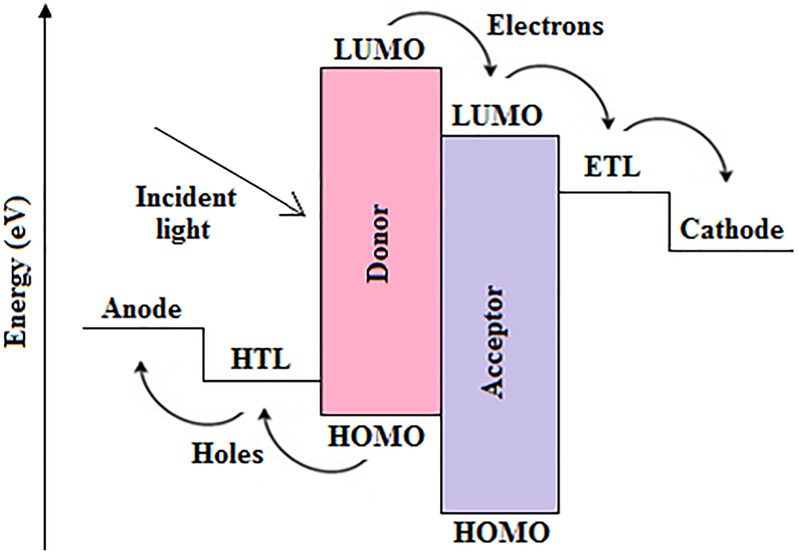
Schematic energy level diagram of a typical BHJ-OSC.

A typical OSC is composed of the electrodes (cathode and anode), charge transport layers (electron transport layer (ETL) and hole transport layer (HTL)), and the photoactive layer, assembled in the conventional or inverted configurations, as presented elsewhere (Muchuweni et al., 2020a). In an OSC, photons from incident solar radiation are transmitted through the substrate, bottom electrode and charge transport layer, so that they reach the photoactive layer, where they are absorbed by the donor material, in which excitons, i.e., strongly bound electron-hole pairs, are generated (Jeon et al., 2017; Ramasamy et al., 2019; Wang et al., 2020; Kang et al., 2021) and localized owing to the large exciton binding energy in the polymer matrix (Hatton et al., 2008). The photogenerated excitons subsequently diffuse within their limited diffusion distance to the interface between materials with dissimilar electron affinities and ionization potentials, i.e., between the donor-acceptor interface, where they are absolutely separated into free charge carriers after overcoming the binding energies (Tessema Mola et al., 2018). However, since the exciton diffusion length in OSCs is small, a bulk heterojunction (BHJ) should exist within the short diffusion distance so that excitons can always reach the donor-acceptor interface for charge separation to occur, and a continuous interpenetrating channel should exist for transporting charge carriers to the electrodes (Jeon Y.-J. et al., 2018; Ramasamy et al., 2019). Actually, the charge carriers are separated into electrons and holes in the acceptor’s lowest unoccupied molecular orbital (LUMO) and donor’s highest occupied molecular orbital (HOMO) levels, respectively, as illustrated in Figure 1. Hence, for the efficient transportation of electrons from the LUMO via the ETL to the cathode, the LUMO level of the acceptor should match well with the ETL’s work function (Huang et al., 2019). Similarly, for the efficient transportation of holes through the HTL to the anode, the donor HOMO level should be well-matched with the HTL’s work function (Huang et al., 2019). Thus, free electrons are transported to the cathode via the ETL, whereas holes are transported in the opposite direction to the anode via the HTL under internal electric fields, which lead to photocurrent generation (Amollo et al., 2018). Finally, the electrodes allow the flow of the photogenerated current to and from the external circuit so that the cell can power a given load.

Despite having a lower projected cost of < $0.07/Wp relative to < $0.35/Wp for commercially available silicon solar cells (Riede et al., 2021), the power conversion efficiency (PCE) of state-of-the-art OSCs (18–25%) (Cho et al., 2020; PV-Magazine, 2020; Salim et al., 2020) is still lower than that of commercially available silicon-based solar cells (above 26%) (Andreani et al., 2018). In addition, when compared with silicon solar cells, OSCs suffer from poor long-term environmental stability, which limits their commercialization (Chen, 2019; Burlingame et al., 2020; Duan and Uddin, 2020; Wang et al., 2021). Hence, this has prompted significant research interest in developing highly efficient and sustainable devices through approaches, such as incorporating novel materials into the different components of OSCs, to overcome the limitations of the commonly used traditional materials.

In this respect, carbon-based materials, such as graphitic carbon nitride, carbon quantum dots, carbon nanotubes (CNTs) and graphene (Jeon et al., 2018b; Nguyen et al., 2019; Ouyang, 2019; Hu et al., 2020; Pan et al., 2020; Qin et al., 2020; Subramanyam et al., 2020; Shin et al., 2021; Vercelli, 2021), have attracted considerable research attention due to their unique physicochemical properties, low-cost, natural abundance of carbon, non-toxicity and compatibility with large-scale solution synthesis (Delacou et al., 2017). Among these, CNTs are more appealing owing to their large specific surface area, tunable band gap, high optical transmittance in the visible region, competitive electrical conductivity, high charge carrier mobility, excellent flexibility and superior mechanical, thermal and chemical stability (Khan et al., 2018; Oseni et al., 2018).

**FIGURE 2:**
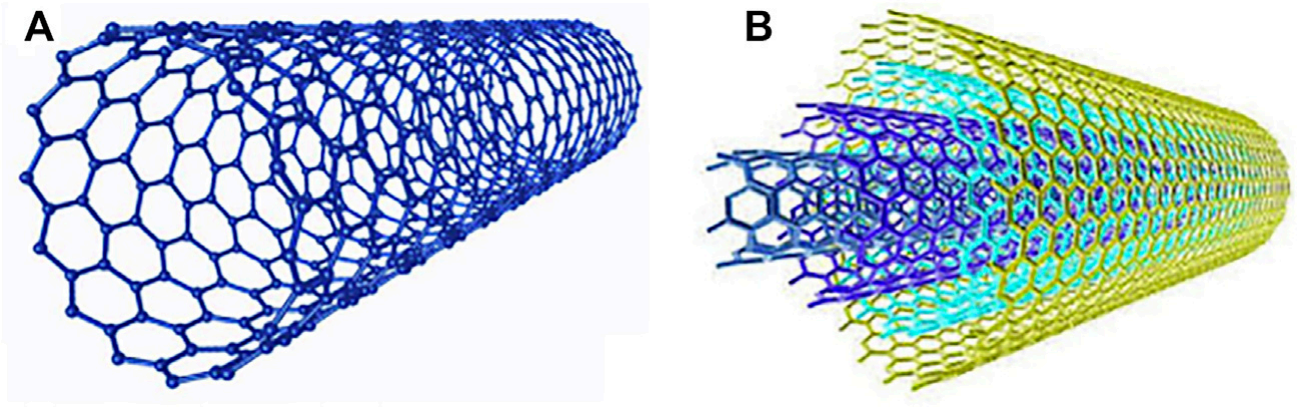
The structure of **(A)** SWCNTs and **(B)** MWCNTs. Adapted with permission (Ouyang, 2019). Copyright 2019, Nano Materials Science.

CNTs, one of the stiffest and strongest materials ever discovered, consist of a cylindrical nanostructure of hexagonally oriented carbon atoms as shown in Figure 2, and can be classified as either semiconducting or metallic depending on their length, diameter and arrangement of hexagonal rings (Alturaif et al., 2014). Also, how graphene layers are wrapped to form a nanotubular morphology, i.e., the chirality of the tubes, significantly determines the electrical properties of the CNTs (Alturaif et al., 2014). CNTs that consist of a single round roll of graphene with a typical diameter of around 0.4–10 nm are referred to as single-walled CNTs (SWCNTs), whereas those consisting of two or more rolled layers of graphene sheets with a typical diameter of 1.4–100 nm are called double-walled CNTs (DWCNTs) and multiwalled CNTs (MWCNTs), respectively (Hatton et al., 2008; Agbolaghi, 2019b; Nguyen et al., 2019). Currently, SWCNTs have been relatively more studied than MWCNTs owing to the novel properties of SWCNTs, such as their band gap energy that can be tuned from 0 to ∼2 eV, thereby varying their electrical conductivity. In addition, the diameter of SWCNTs falls within the preferred range of up to 20 nm for OSC applications since the thickness of the organic layer in OSCs is typically up to 200 nm (Hatton et al., 2008). Hence, SWCNTs exhibit unusual properties of either semiconducting or metallic materials, whereas MWCNTs are zero band gap materials with metallic properties (Obaidullah et al., 2018; Ouyang, 2019).

CNTs have been prepared by means of several techniques, such as chemical vapour deposition (CVD), laser ablation and arc discharge (Jeon et al., 2018c; Khan et al., 2021; Shen, 2021). Among these, CVD is more appealing owing to its potential for large-scale synthesis. Also, CNTs can be deposited onto various components of OSCs by using different techniques, such as spray coating, dip coating, spin coating, sputtering and CVD (Khan et al., 2018). However, the insolubility in organic solvents, entanglement and poor alignment of CNTs, in addition to the presence of metal impurities, are the main limitations for the incorporation of CNTs into various layers of OSCs. These limitations cause unfavourable short-circuits, surface charge carrier trapping and reduction in charge carrier mobility, thereby increasing leakage current and recombination pathways (Oseni et al., 2018). Although sonication has been employed to disperse CNTs, it often causes them to break and alter their properties; hence, covalent and non-covalent functionalization have been developed in an attempt to improve the dispersibility of CNTs (Tasis et al., 2006; Hadi et al., 2020; Khan et al., 2021). Among these functionalization techniques, covalent functionalization involves the use of chemical reactions to introduce various functional groups onto the surfaces of CNTs (Nan et al., 2016). Nonetheless, the harsh chemical reactions impair the π-bonds of CNTs and degrade the CNTs’ intrinsic electrical, mechanical and thermal properties (Nan et al., 2016).

On the other hand, non-covalent functionalization involves the adsorption of dispersants onto the surface of CNTs through non-covalent interactions, such as interactions between the π-electronic systems of CNTs and dispersants, which break the van der Waals forces between the individual CNTs, without any disruptions to the CNTs’ π-electron conjugated structure (Keru et al., 2014). This, in turn, improves the interaction of CNTs with other constituent materials that make up the different components of OSCs, thereby increasing charge carrier mobility (An et al., 2017; Hadi et al., 2020). This also addresses the challenge of leakage current due to short-circuits that usually originate from the bundling of poorly dispersed CNTs. In addition, magnetic or electric fields may be applied, or the CNTs may be grown along a single direction to improve their alignment (Khan et al., 2021), thereby minimizing undesirable short-circuits that usually emanate from poorly aligned CNTs, and facilitating the efficient transportation of charge carriers, which subsequently leads to a substantial improvement in device performance.

However, the PCE of CNT-based OSCs is still relatively lower than that of OSCs based on traditional materials; hence, the optoelectronic properties of CNTs need further optimization for them to produce devices that can compete with those based on their traditional counterparts. Also, recent applications of carbonaceous nanostructures, especially CNTs, to improve the efficiency and sustainability of BHJ-OSCs for possible commercialization are yet to be satisfactorily reported. Therefore, this review focuses on the recent applications of CNTs, mainly over 2018–2021, in the electrodes, active layers and charge transport layers of OSCs, to improve not only the PCE, but also to enhance the long-term operational stability, thereby paving the way for the commercial application of OSCs. In addition, the merits, limitations and outlook for the future fabrication of high performance and sustainable OSCs are discussed.

## Transparent Conducting Electrode

The most widely used transparent conducting electrode is indium tin oxide (ITO) due to its high optical transparency and electrical conductivity (Zhang Y. et al., 2020; Du et al., 2021), which facilitate the entrance of more light into the cell, and the efficient collection and transportation of photogenerated charge carriers to the external circuit. However, indium is an expensive and toxic rare earth metal, which limits the sustainability of ITO (Muchuweni et al., 2016a; Muchuweni et al., 2016b; Muchuweni et al., 2017a; Muchuweni et al., 2017b). Also, ITO is brittle; hence, not compatible with flexible substrates, and has instability issues in acidic and basic environments, which lead to device degradation (Jeon et al., 2015; Patil et al., 2021). In addition, good quality ITO is usually prepared using complicated, expensive and high-temperature vacuum-based processes (Lu et al., 2017; Muchuweni et al., 2020b).

Therefore, considerable effort has been exerted on developing alternative transparent conducting electrodes by using novel materials, such as metal grids (Mo et al., 2016; Jeong et al., 2018), silver nanowires (AgNWs) (Sun et al., 2019; Arulkumar et al., 2021), CNTs (Yu et al., 2016; Jeon et al., 2017) and graphene (Jeon et al., 2017; Keyvani-Someh et al., 2017), owing to their competitive optoelectronic properties. Among these, CNTs are more appealing due to their low-cost, easy availability of raw materials, low-temperature solution processability, large specific area, flexibility, stability, and capability of offering a good balance between high optical transmittance in the visible region and low sheet resistance (Lu et al., 2017; Matsuo, 2021; Shen, 2021). In addition, the work function of CNTs is comparable to that of ITO, which reduces the energy barrier; hence, facilitating the efficient collection of photogenerated charge carriers at the electrode.

Although CNTs have been recently employed to replace or modify the commonly used ITO anode in conventional OSCs, relatively few or no detailed studies have reported their application as the cathode, mainly due to the relatively higher work function of CNTs, as compared with the low work function of the widely used ETLs, which increases the potential barrier, thereby limiting the collection of electrons at the cathode. Thus, for the cathode to efficiently collect and transport electrons, its work function should be relatively lower than that of the ETL (Muchuweni et al., 2020a); hence, making the high work function of CNTs not suitable for cathode applications. Therefore, this section only reviews the recent application of CNTs in the anode of BHJ-OSCs.

### Anode

As mentioned earlier, CNTs have recently gained tremendous research attention as promising alternatives to the commonly used ITO bottom electrodes, i.e., anodes in conventional OSCs. Nevertheless, CNT-based electrodes still exhibit relatively higher sheet resistances of ∼100 Ω sq^−1^ at a transparency of 85% in the visible region when compared with the required sheet resistance of ∼10 Ω sq^−1^ at 85% transparency (Yang and Lee, 2020), thereby giving rise to devices with relatively low efficiency. Hence, as a future research direction, a substantial reduction in the sheet resistance of CNTs, while maintaining high optical transparency, is required.

On the other hand, the most commonly used anode materials in inverted OSCs, i.e., top electrodes, are the high work function metals, such as Ag or Au (Ali et al., 2018). However, these metal electrodes are opaque and reflective. Moreover, they are usually deposited under high vacuum and temperature conditions using thermal evaporation, which is complicated and expensive, thereby limiting the facile fabrication of low-cost and sustainable devices (Zhang et al., 2018; Kumar et al., 2020). In addition, Ag has stability issues, and Au is expensive (Lee et al., 2018). Therefore, there has been significant research interest in developing alternative materials with comparable performance, of which CNTs are more attractive due to their low-cost, facile fabrication procedures, competitive optoelectronic properties and excellent stability.

**FIGURE 3:**
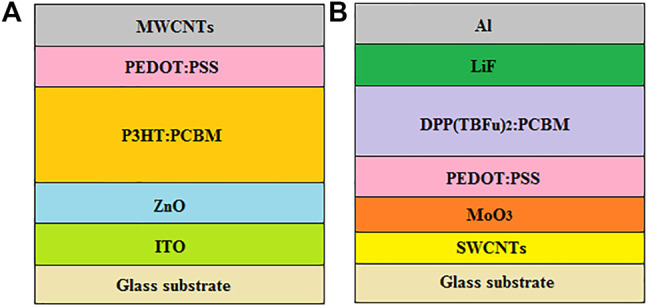
Schematic diagram of **(A)** an inverted OSC based on a MWCNT anode and **(B)** a conventional OSC based on a SWCNT anode.

In this respect, MWCNTs have been employed as anodes in inverted OSCs based on an ITO cathode, zinc oxide (ZnO) ETL, poly (3,4-ethylenedioxythiophene):poly (styrenesulfonate) (PEDOT:PSS) HTL and poly (3-hexylthiophene) [6,6]-phenyl-C_61_-butyric acid methyl ester (P3HT:PC_61_BM) active layer (Ali et al., 2018), as illustrated in Figure 3A. The high optical transmittance of MWCNTs (85% for a single sheet) permitted the passage of more light to the active layer, which significantly improved photon harvesting; hence, promoting the generation of excitons. In addition, the low sheet resistance of MWCNTs (149 Ω sq^−1^) facilitated the efficient collection and transportation of holes from the HTL to the anode, thereby increasing the J_sc_. This resulted in devices with an optimum PCE of 1.46%, which was comparable to 2.25% for the Ag anode-based reference cell. Interestingly, the MWCNT-based devices managed to retain above 80% of their original PCE after storage for 10 days under environmental conditions, and also managed to maintain their initial PCE, above 90%, after 100 bending cycles, demonstrating their superior long-term environmental stability and excellent flexibility. Recently, a relatively high PCE of 7% has been obtained after introducing Ag nanoparticles (NPs) into MWCNT-based anodes of inverted OSCs (Zhang et al., 2021), demonstrating the significance of nanocomposites towards enhancing the efficiency of devices. In similar studies, but with conventional OSCs based on MWCNTs (Mugadza et al., 2017) and DWCNTs (Zhang et al., 2018), as anodes, more stable devices with optimum PCEs of 0.68 and 1.71%, respectively, were fabricated. This demonstrates the ample potential of CNTs as low-cost alternative electrodes for the future fabrication of highly efficient, stable and flexible devices.

SWCNTs have also been employed as anodes in conventional OSCs with the SWCNTs/molybdenum trioxide (MoO_3_)/PEDOT:PSS/3,6-bis [5-(benzofuran-2-yl)thiophen-2-yl]-2,5-bis(2-ethylhexyl)pyrrolo [3,4-c]pyrrole-1,4-dione (DPP(TBFu)_2_):PCBM/lithium fluoride (LiF)/aluminium (Al) configuration, as illustrated in Figure 3B, which exhibited a best PCE of 1.9% (Delacou et al., 2017). However, this PCE was lower than 4.4% for the control devices based on ITO anodes, which was attributed to the lower J_sc_ of 3.16 mA cm^−2^ for the SWCNT-based devices, when compared with 9.38 mA cm^−2^ for the ITO reference devices. This was probably due to the relatively lower electrical conductivity of SWCNTs than that of ITO, emanating from the relatively higher series resistance of SWCNTs (40 Ω cm^2^) than 22 Ω cm^2^ for ITO. In addition, the SWCNT-based devices had a relatively lower shunt resistance of 2.2 × 10^4^ Ω cm^2^, in comparison with 1.7 × 10^6^ Ω cm^2^ for the ITO control devices, which indicated the presence of significant charge carrier recombination, resulting in a lower FF; and, hence, giving rise to poor device performance.

**FIGURE 4:**
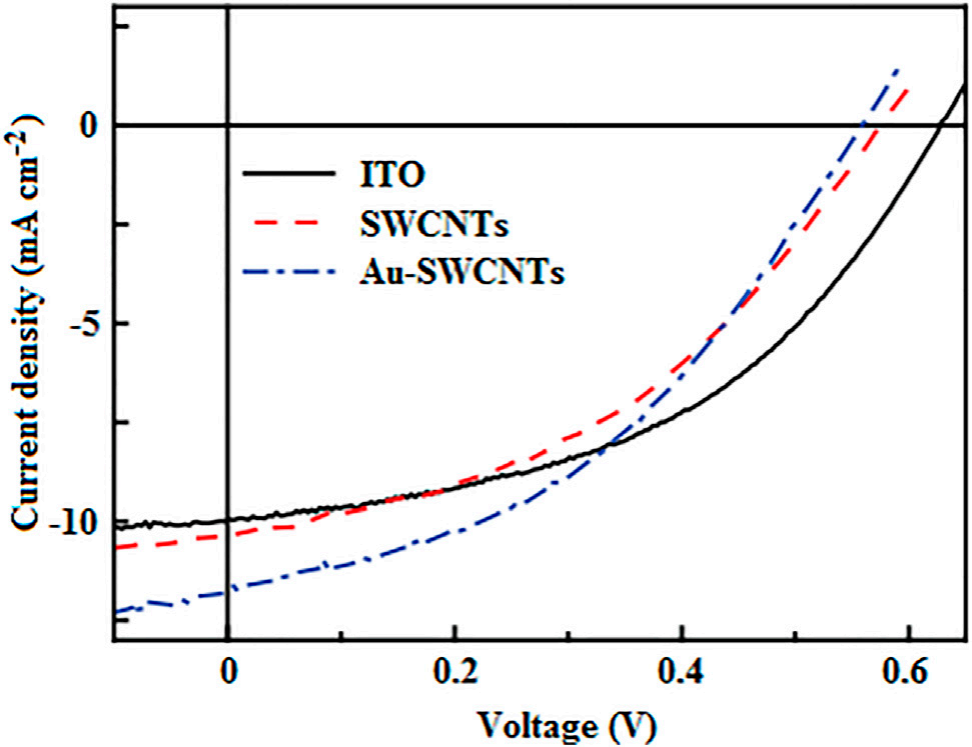
J-V characteristics of conventional OSCs employing ITO, SWCNTs, and Au-SWCNTs as anodes. Adapted with permission (Fan et al., 2017). Copyright 2017, Chinese Physical Society and Institute of Physics.

A further improvement was made by doping SWCNTs with Au, which reduced the sheet resistance from 182 Ω sq^−1^ at a transmittance of 85% for the pristine SWCNTs to 60 Ω sq^−1^ at 82% transmittance (Fan et al., 2017). The resulting Au-SWCNTs were employed as anodes in conventional OSCs, which displayed the best PCE of 2.74%. This outperformed the pristine SWCNT-based device with a PCE of 2.52% and was comparable to 2.93% for the ITO reference device. The relatively poor performance of pristine SWCNT-based devices was attributed to their slightly low V_oc_, emanating from short-circuits due to protruding SWCNTs. Also, the relatively low optical transmittance of the as-prepared SWCNTs usually obstructs the passage of more light to the active layer, resulting in low photon absorption and poor exciton generation, which impair device performance. In addition, the relatively high sheet resistance of pristine SWCNTs often leads to high series resistance, which restricts the efficient flow of holes to the anode, thereby giving rise to a low J_sc_, as shown in Figure 4, and hence results in low device efficiency.

In a similar study, SWCNTs were doped with tetrafluoroethylene-based fluoropolymer-copolymer sulfonic acid (TFES), trifluoromethanesulfonic acid (TFMS) and nitric acid (HNO_3_), and employed as anodes in conventional OSCs, which exhibited PCEs of 8.0, 8.3, and 8.5%, respectively (Jeon et al., 2018a). This outperformed the pristine SWCNT-based device with a PCE of 4.4%, and was comparable to the ITO reference device with a PCE of 9.1%. The relatively low efficiency of the as-prepared SWCNT-based devices was attributed to their relatively high series resistance (60 Ω cm^2^) and low shunt resistance (320 Ω cm^2^), resulting in low J_sc_ and FF, due to poor hole transport and high charge carrier recombination. In addition, the devices based on pristine SWCNTs, TFES-SWCNTs, TFMS-SWCNTs and HNO_3_-SWCNTs, respectively, managed to retain above 70, 87, 63, and 41% of their initial PCE after storage for 60 days in a dark and N_2_ environment, demonstrating their excellent long-term stability. Therefore, as a future research direction, the chemical doping of CNT-based electrodes can be employed to yield highly efficient and sustainable devices.

Recently, nanocomposites of SWCNTs and AgNWs with a low sheet resistance of 50 Ω sq^−1^ and a high average optical transmittance of 94% in the visible region have also been used as anodes in conventional OSCs, which exhibited a maximum PCE of 2.21% (Yang and Lee, 2020). This outperformed the pristine AgNW-based devices with a PCE of 1.43%, mainly due to the relatively high sheet resistance of the pristine AgNWs of 154 Ω sq^−1^, giving rise to low electrical conductivity, and hence poor hole transport. Also, the root mean square surface roughness (R_rms_) of 13.5 nm for the smooth SWCNT/AgNW composite electrode, which was relatively lower than 23.8 nm for the rough pristine AgNWs, resulted in a relatively high shunt resistance, thereby suppressing charge carrier recombination and leakage current when compared with pristine AgNWs that had a low shunt resistance; and hence, were prone to significant charge carrier recombination and leakage current, which subsequently reduce the device’s efficiency.

Table 1 summarizes the photovoltaic parameters of OSCs based on CNT anodes discussed in this work. Interestingly, the best PCE of 8.5% was obtained when HNO_3_-SWCNTs were used as the anode (Jeon et al., 2018a). Therefore, the chemical doping of CNTs is one of the best techniques that can be employed in future research to produce a substantial improvement in the optoelectronic properties and stability of the anode, thereby opening up avenues for the future realization of low-cost, flexible, highly efficient and sustainable carbon-based devices.

**TABLE 1:** Photovoltaic parameters of OSCs employing CNT-based anodes.

Anode	V_oc_ (V)	J_sc_ (mA cm^−2^)	FF	PCE (%)	References
MWCNTs	0.57	7.53	0.34	1.46	Ali et al. (2018)
AgNPs/MWCNTs	0.76	15.23	0.60	7.00	Zhang et al. (2021)
MWCNTs	0.17	15.62	0.26	0.68	Mugadza et al. (2017)
DWCNTs	0.50	10.90	0.32	1.71	Zhang et al. (2018)
SWCNTs	0.80	3.20	0.40	1.90	Delacou et al. (2017)
SWCNTs	0.58	10.40	0.42	2.52	Fan et al. (2017)
Au-SWCNTs	0.56	11.70	0.42	2.74	Fan et al. (2017)
SWCNTs	0.80	12.00	0.46	4.40	Jeon et al. (2018a)
TFES-SWCNTs	0.80	14.30	0.70	8.00	Jeon et al. (2018a)
TFMS-SWCNTs	0.81	14.10	0.73	8.30	Jeon et al. (2018a)
HNO_3_-SWCNTs	0.81	14.20	0.74	8.50	Jeon et al. (2018a)
SWCNTs/AgNWs	0.61	7.60	0.48	2.21	Yang and Lee (2020)

## Active Layer

The active layer of BHJ-OSCs is composed of an intimate blend of electron donor and acceptor materials, which does not only absorb light, generate excitons and provide multiple sites with large donor-acceptor interfacial area for effective dissociation of charge carriers, but also removes the requirement for long exciton diffusion lengths, and provides percolation pathways for efficient charge carrier transport (Oseni and Mola, 2017; Hou et al., 2018; Rafique et al., 2018; Gusain et al., 2019; Wu et al., 2019; Chen et al., 2020; Song et al., 2020; Xu et al., 2021). However, the photoactive layer often has issues, such as low optical absorption in the visible range, the need for more energy to dissociate the strongly bound photogenerated excitons, the presence of defects and charge carrier traps, short lifetime of charge carriers owing to recombination, discontinuous pathways for charge carrier transport, poor charge carrier mobility due to the hopping transport mechanism, and long-term instability due to degradation of the active layer materials (Paul et al., 2017; Subramanyam et al., 2020). Hence, traditional active layer materials need to be replaced or modified; to broaden the absorption spectrum for effective photon absorption and exciton generation; to increase the donor-acceptor interfacial area for significant exciton dissociation; to provide additional conductive networks for efficient charge carrier transport; and to prevent air and moisture penetration for improving long-term stability.

The most commonly used donor and acceptor materials are the p-type semiconducting polymer, P3HT, and fullerene, PCBM, respectively (Lim et al., 2018; Rathore et al., 2018; Dlamini et al., 2020; Gao et al., 2020; Milanovich et al., 2020; Ghosekar and Patil, 2021). This is due to the merits of P3HT, such as outstanding solubility in organic solvents, high absorption in the visible region, enhanced crystallinity, excellent charge carrier mobility and high stability (Khanh et al., 2020). In addition, PCBM has an excellent electron accepting capability and forms suitable nanoscale morphological networks with P3HT (Sivakumar et al., 2020). However, PCBM suffers from drawbacks, such as high-cost, low electrical conductivity, low charge carrier mobility, limited energy level engineering, weak optical absorption in the visible region, inferior mechanical flexibility, poor air and thermal stability, and complicated synthesis procedures (Amollo et al., 2017; Aïssa et al., 2019; Liang et al., 2019; Sivakumar et al., 2020; Zhao et al., 2020). Therefore, to address the aforementioned limitations, recent studies have focussed on replacing or modifying PCBM with carbon-based materials, particularly CNTs, owing to their excellent electron-accepting capability, large specific surface area, broad absorption spectrum, low reflectance, high charger carrier mobility, facile tunability of band gap, superior stability, and low-cost (Bhatia and Kumar, 2017; Fraga Domínguez et al., 2017). Furthermore, the band offset of CNTs and the donor polymer, and high built-in electric field at the polymer-CNT interface, have the potential to enhance the dissociation of excitons at the donor-acceptor interface, thereby facilitating the efficient transfer of electrons from the polymer to the CNTs (Fraga Domínguez et al., 2017; Agbolaghi, 2019b).

**FIGURE 5:**
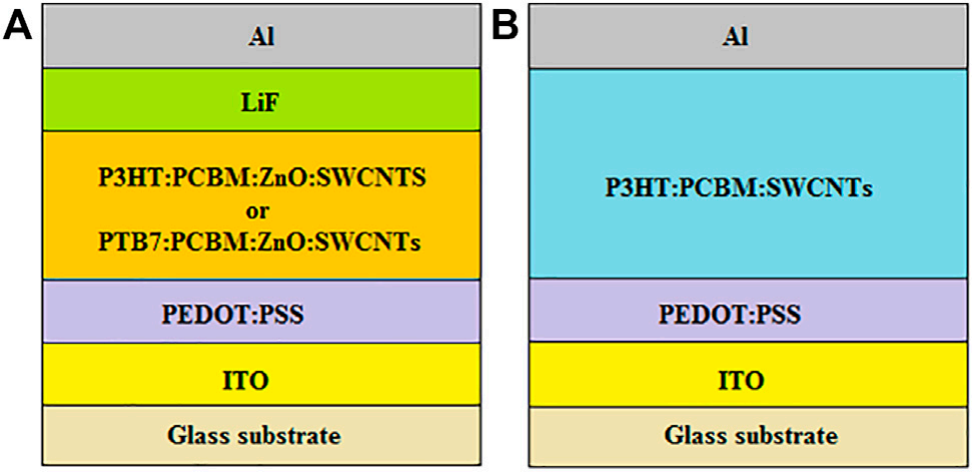
Schematic diagram of conventional BHJ-OSCs with **(A)** ZnO:SWCNTs and **(B)** SWCNTs in the active layers.

Being motivated by this, ZnO:SWCNTs have been incorporated into the polythieno [3,4-b]thiophene-co-benzodithiophene (PTB7):PCBM and P3HT:PCBM active layer blends of conventional BHJ-OSCs (Oseni et al., 2018), as illustrated in Figure 5A. Increasing the ZnO:SWCNT concentration to 6 wt% improved the generation of excitons and enhanced the interpenetrating networks for both hole and electron transport, which increased the J_sc_ and FF, resulting in relatively higher PCEs of 4.66 and 3.10% for the devices based on PTB7:PCBM:ZnO:SWCNTs and P3HT:PCBM:ZnO:SWCNTs, respectively, when compared with 2.76 and 1.92% for the pristine PTB7:PCBM and P3HT:PCBM devices, respectively. However, the introduction of excessive concentrations of ZnO:SWCNTs into the active layer led to a decrease in the J_sc_ and FF, probably due to the high series resistance and low shunt resistance, originating from the agglomeration and bundling of nanotubes, which produces charge carrier traps that prevent the smooth flow of charge carriers, resulting in high charge carrier recombination, as well as causing high leakage current due to short-circuits, thereby impairing device performance.

SWCNTs have also been incorporated into the P3HT:PCBM active layer blend of BHJ-OSCs (Kadem et al., 2018; Aïssa et al., 2019) as illustrated in Figure 5B. This resulted in devices with optimum PCEs of 2.20 and 3.54%, which outperformed their pristine P3HT:PCBM-based counterparts that had PCEs of 1.52 and 1.17%, respectively. This was attributed to the synergy between the electron-accepting nature of the fullerene and the rapid transportation of electrons through the additional percolation pathways created by the SWCNTs, which promoted the effective separation and transportation of charge carriers, thereby increasing the photogenerated current. In addition, the low R_rms_ of the SWCNT-based active layers of less than 5 nm, demonstrated that the hybrid active layer films were smooth and homogeneous; hence, prone to fewer short-circuits and few charge carrier traps, resulting in low leakage current and less recombination, which improves electron transport and subsequently increases the devices’ PCE. A further increase in PCE from 2.20 to 2.32% was observed after incorporating acid-treated SWCNTs into the P3HT:PCBM blend (Kadem et al., 2018), which was ascribed to the increase in electrical conductivity from 130 mS cm^−1^ for the P3HT:PCBM blend with non-treated SWCNTs to 230 mS cm^−1^ for the P3HT:PCBM blend with acid-treated SWCNTs.

A further improvement was made by introducing MWCNTs:rGO into the P3HT:PCBM active layer blend of BHJ-OSCs, which resulted in more stable devices with a relatively higher PCE of 4.13% when compared with 2.91% for the pristine P3HT:PCBM devices (Mahakul et al., 2019). This was ascribed to the introduction of MWCNTs:rGO as additives to P3HT:PCBM, which not only increased the optical absorbance and donor-acceptor interfacial area for effective photon harvesting, exciton generation and exciton dissociation, but also provided smooth conductive pathways for the efficient transportation of photogenerated charge carriers and acted as protective layers against air and moisture penetration; hence, improving device performance and stability.

**FIGURE 6:**
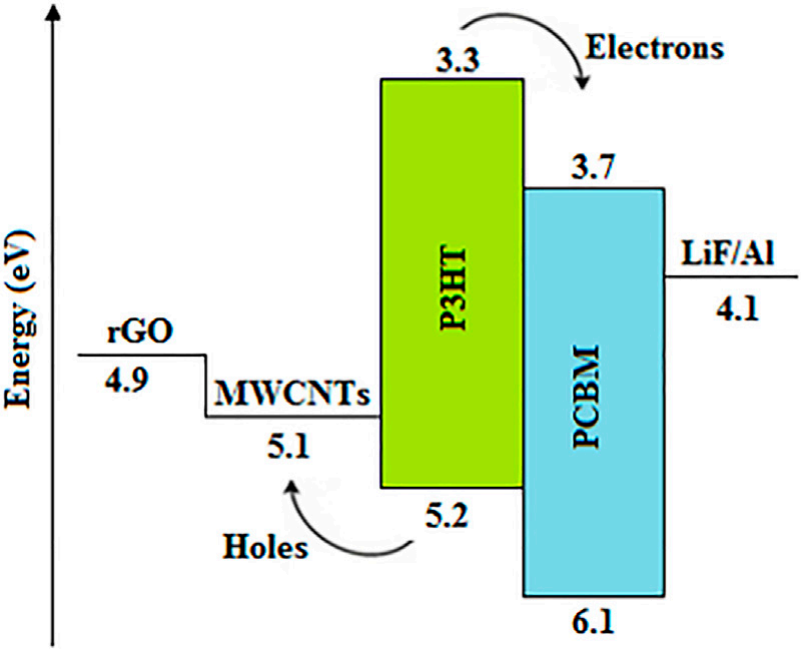
Schematic energy level diagram of the P3HT:PCBM:MWCNT active layer-based OSC.

MWCNTs have also been integrated with the P3HT:PCBM active layer blend of OSCs (Khanh et al., 2020; Subramanyam et al., 2020; Khan et al., 2021), which significantly enhanced the optical absorption and created large donor-acceptor interfaces for improving the generation and dissociation of excitons. Also, the relatively higher work function of the MWCNTs of ∼5.1 eV provided proper energy level alignment for the fast extraction and transportation of holes from the polymer blend to the MWCNTs, in addition to the faster electron transport by PCBM as illustrated in Figure 6, which played a substantial role in suppressing charge carrier recombination, thereby increasing the FF and J_sc_, resulting in devices with relatively higher PCEs of 2.35 (Khanh et al., 2020), 1.88 (Khan et al., 2021), and 4.86% (Subramanyam et al., 2020), when compared with the pristine P3HT:PCBM-based devices. Nevertheless, extremely high concentrations of MWCNTs reduced the J_sc_ and FF, which in turn impaired the device performance due to the agglomeration of MWCNTs as their quantity increased, giving rise to leakage current through short-circuits. Furthermore, the agglomeration of CNTs often reduces the surface area available for the formation of heterojunctions, thereby reducing exciton generation; hence, significantly reducing the photogenerated current, and subsequently giving rise to devices with low PCE (Mahakul et al., 2019).

Recently, MWCNTs grafted with poly (3-dodecylthiophene) (MWCNTs-*graft*-PDDT) were crystallized with P3HT (Agbolaghi, 2019b; Hadi et al., 2020) and poly[bis(triiso-propylsilylethynyl) benzodithiophene-bis(decyltetradecylthien) naphthobisthiadiazole] (PBDT-TIPS-DTNT-DT) (Agbolaghi, 2019a), and the resulting nanohybrids were integrated with P3HT:PC_71_BM (Agbolaghi, 2019b; Hadi et al., 2020) and PBDT-TIPS-DTNT-DT:PC_71_BM (Agbolaghi, 2019a) active layer blends of BHJ-OSCs. Interestingly, the surface modification of MWCNTs helped to increase their dispersion in organic solvents, which enhanced the interaction between the P3HT and MWCNT phases. This, in turn, reduced the agglomeration of MWCNTs, and hence reduced short-circuits, surface charge trapping and recombination, resulting in devices with relatively higher PCEs of 5.40 (Agbolaghi, 2019b) and 4.18% (Hadi et al., 2020), respectively, when compared with 2.13 and 1.14% for the corresponding unmodified MWCNT-based devices. Also, the larger specific surface area and larger crystallite sizes of MWCNTs-*graft*-PDDT/P3HT with fewer grain boundaries and better crystallinity, respectively, increased the donor-acceptor interfacial area and formed conductive pathways, which increased the exciton dissociation rate and charge carrier mobility, resulting in high J_sc_, thereby improving the device performance. However, the grafting of MWCNTs to PDDT weakened the assembling and crystallization of the PBDT-TIPS-DTNT-DT chains onto the MWCNTs, which reduced the PCE from 4.07 to 3.69% for the unmodified MWCNT- and the grafted MWCNT-based devices, respectively (Agbolaghi, 2019a).

Table 2 summarizes the photovoltaic parameters of OSCs with CNT-based active layers. From the reviewed reports, the highest PCE of 5.40% was observed in P3HT:PCBM:MWCNTs-*graft*-PDDT/P3HT active layer-based devices (Agbolaghi, 2019b). This demonstrates the excellent potential of CNTs as future additives of choice to the P3HT:PCBM active layer blend, rather than as substitutes to PCBM, capable of increasing not only the donor-acceptor interfacial area for the effective dissociation and extraction of excitons, but also providing smooth conductive pathways for the efficient transportation of photogenerated charge carriers, and as protective layers against air and moisture penetration; hence, improving device performance and stability.

**TABLE 2:** Photovoltaic parameters of OSCs employing CNT-based active layers.

Active layer	V_oc_ (V)	J_sc_ (mA cm^−2^)	FF	PCE (%)	References
P3HT:PCBM:ZnO:SWCNTs	0.57	11.22	0.49	3.10	Oseni et al. (2018)
PTB7:PCBM:ZnO:SWCNTs	0.75	14.40	0.43	4.66	Oseni et al. (2018)
P3HT:PCBM:SWCNTs	0.55	7.34	0.55	2.20	Kadem et al. (2018)
P3HT:PCBM:acid-treated SWCNTs	0.54	8.00	0.54	2.32	Kadem et al. (2018)
P3HT:PCBM:SWCNTs	0.66	9.95	0.54	3.54	Aïssa et al. (2019)
P3HT:PCBM:MWCNTs:rGO	0.65	11.01	0.50	4.13	Mahakul et al. (2019)
P3HT:PCBM:MWCNTs	0.87	5.05	0.54	2.35	Khanh et al. (2020)
P3HT:PCBM:MWCNTs	0.49	8.64	0.44	1.88	Khan et al. (2021)
P3HT:PCBM:MWCNTs	0.67	11.81	0.62	4.86	Subramanyam et al. (2020)
P3HT:PCBM:MWCNTs	0.61	8.18	0.42	2.13	Agbolaghi (2019b)
P3HT:PCBM:MWCNTs-*graft*-P3HT	0.61	11.99	0.56	4.11	Agbolaghi (2019b)
P3HT:PCBM:MWCNTs-*graft*-PDDT/P3HT	0.63	13.11	0.65	5.40	Agbolaghi (2019b)
P3HT:PCBM:MWCNTs-*graft*-PDDT/P3HT	0.62	11.25	0.60	4.18	Hadi et al. (2020)
P3HT:MWCNTs-*graft*-PDDT/P3HT	0.63	9.69	0.52	3.17	Hadi et al. (2020)
P3HT:PCBM:MWCNTs/P3HT	0.65	8.78	0.48	2.74	Hadi et al. (2020)
P3HT:MWCNTs/P3HT	0.64	4.56	0.39	1.14	Hadi et al. (2020)
PBDT-TIPS-DTNT-DT:PCBM:MWCNTs/PBDT-TIPS-DTNT-DT	0.69	10.17	0.58	4.07	Agbolaghi, (2019a)
PBDT-TIPS-DTNT-DT:PCBM:MWCNTs-*graft*-PDDT/PBDT-TIPS-DTNT-DT	0.68	9.51	0.57	3.69	Agbolaghi, (2019a)

## Charge Transport Layer

The extraction and transportation of charge carriers from the active layer to the electrodes of OSCs have been significantly improved by introducing charge transport layers that reduce the energy barrier and prevent the direct contact between the active layer and electrodes for the effective extraction and transportation of charge carriers and suppression of recombination (Amollo et al., 2018; Ramasamy et al., 2019; Tian et al., 2021). From this viewpoint, HTLs and ETLs extract and transport holes and electrons from the photoactive layer to the anode and cathode, respectively, while selectively blocking electrons (for HTLs) and holes (for ETLs), thereby suppressing charge carrier recombination. In addition, the charge transport layers are usually transparent to allow the incoming photons to reach the active layer for exciton generation. Also, the charge transport layers improve the stability of devices by forming protective layers that seal the active layer materials from the diffusion of moisture and air (Rafique et al., 2018; Amusan et al., 2019; Liu et al., 2019). Therefore, the charge transport layers play a substantial role in determining the overall efficiency and stability of OSCs.

### Hole Transport Layer

HTLs are high work function p-type materials that are responsible for reducing the potential barrier and providing an Ohmic contact at the active layer-anode interface, which improves the extraction and transportation of holes from the active layer to the anode, while selectively blocking electron transport, thereby suppressing charge carrier recombination (Amollo et al., 2018; Jeon Y.-J. et al., 2018). Several HTL materials, including the p-type semiconducting polymer PEDOT:PSS; inorganic metal oxides, e.g., nickel oxide (NiO), MoO_3_, and vanadium pentoxide (V_2_O_5_); and carbon-based materials, e.g., CNTs and graphene, have been recently employed in OSCs (Mbuyise et al., 2017; Singh et al., 2017; Hilal and Han, 2019; Mohammad et al., 2019; Rafique et al., 2019; Li J. et al., 2020). Among these, PEDOT:PSS is the most commonly used HTL due to its high optical transmittance in the visible region, which permits more light to pass to the active layer for effective exciton generation, and its suitable work function, which aligns the energy levels between the active layer and anode for efficient hole extraction (Dang et al., 2018; Zhang W. et al., 2020). In addition, PEDOT:PSS is compatible with low-cost solution processing and has a smooth surface morphology, which moderates the surface roughness of the anode; hence, reducing the likelihood of undesirable effects, such as charge carrier traps and short-circuits, and thereby suppressing charge carrier recombination and leakage current (Ricciardulli et al., 2017; Sorkhishams et al., 2019). Nonetheless, the hygroscopic nature of PEDOT:PSS enables the unfavourable penetration of water into the active layer, and the acidity of PEDOT:PSS causes etching of the ITO anode, which subsequently leads to low device efficiency and poor long-term stability (Subramanyam et al., 2019). Furthermore, PEDOT:PSS has poor hole selectivity, i.e., weak electron blocking capabilities, and the insulating nature of PSS chains causes PEDOT:PSS to have a relatively low electrical conductivity (Dang et al., 2018; Pali et al., 2018; Hayat et al., 2019).

Although inorganic metal oxides have been proposed as potential alternatives to PEDOT:PSS due to their excellent hole collection property, suitable work function, electron-blocking capability and superior stability, their use has been limited by their requirement for high-vacuum and high-temperature deposition equipment (Ricciardulli et al., 2017), which is expensive and complicated to use, incompatible with flexible substrates, and consumes more energy (Zhang et al., 2019; Xu et al., 2020). Hence, to overcome the aforementioned drawbacks, CNTs have been recently reported as one of the most promising alternative HTL materials, capable of modifying or replacing PEDOT:PSS, due to their ballistic charge transport capability, high electrical conductivity, high optical transmittance in the visible range, excellent stability, high flexibility, and solution processability (Subramanyam et al., 2020).

**FIGURE 7:**
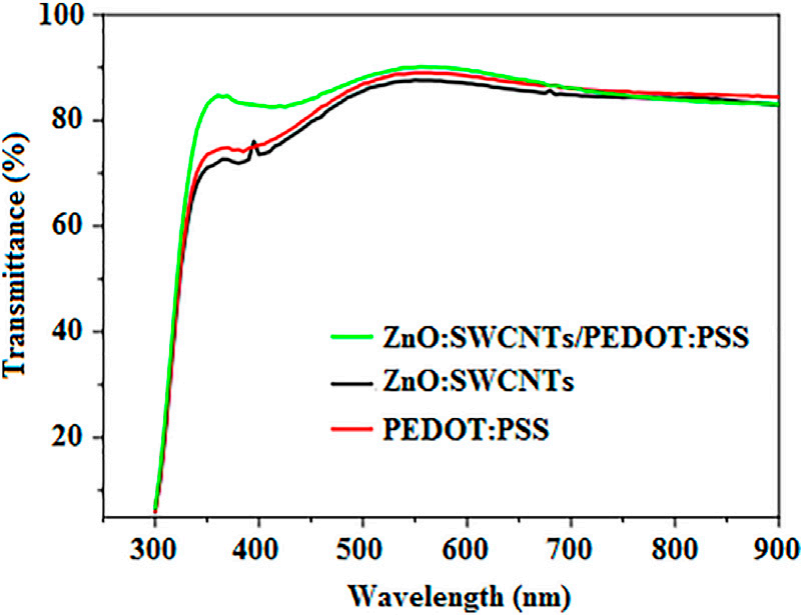
The optical transmittance spectra of ZnO:SWCNTs/PEDOT:PSS, ZnO:SWCNTs and PEDOT:PSS films on ITO-coated glass substrates. Adapted with permission (Mbuyise et al., 2017). Copyright 2017, Elsevier.

Inspired by this, ZnO-doped SWCNTs (ZnO:SWCNTs) have been incorporated into PEDOT:PSS and used as a composite HTL in BHJ-OSCs, resulting in devices with a relatively higher PCE of 4.1% when compared with the pristine PEDOT:PSS reference device that had a PCE of 1.9% (Mbuyise et al., 2017). This was attributed to the high optical transmittance of the ZnO:SWCNTs/PEDOT:PSS HTL in the visible region, as shown in Figure 7, which allowed the passage of more light to the active layer, thereby facilitating significant photon harvesting, leading to effective exciton generation. In addition, the ZnO:SWCNTs reduced the potential barrier between the P3HT:PCBM active layer and the ITO anode, which enhanced the extraction and mobility of holes, and provided better interfacial contact, which lowered the series resistance and facilitated the efficient collection of holes at the ITO anode, resulting in an enhanced J_sc_. Also, the devices with ZnO:SWCNTs loading of 2.5, 5.0, and 10.0 wt% managed to retain 84, 51, and 83% of their original PCE, respectively, after being stored at 100°C without encapsulation in a nitrogen atmosphere, demonstrating their excellent stability.

**FIGURE 8:**
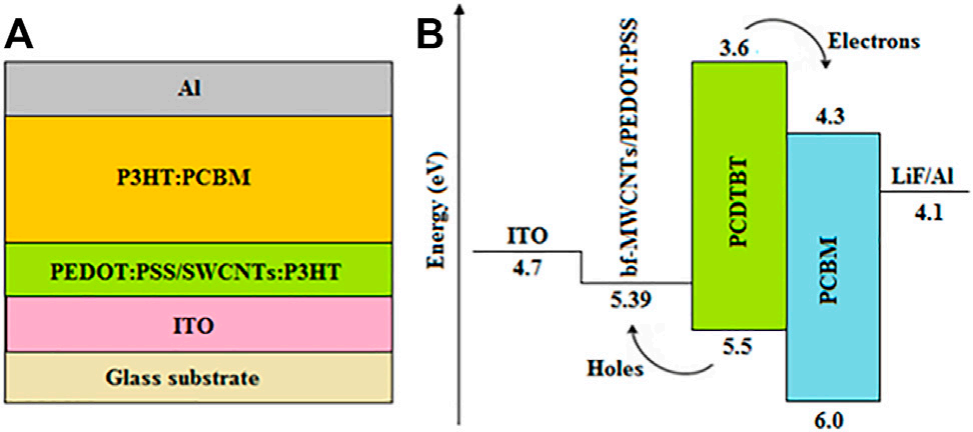
**(A)** Schematic diagram of the PEDOT:PSS/SWCNTs:P3HT-based BHJ-OSC, and **(B)** energy level diagram of the bf-MWCNTs/PEDOT:PSS HTL-based OSC with the poly [N-9′-heptadecanyl-2,7-carbazole-alt-5,5-(4′,7′-di-2-thienyl-2′,1′,3′-benzothiadiazole)] (PCDTBT):PCBM active layer blend.

In another study, acid-treated SWCNTs have been integrated with P3HT and the resulting hybrids were incorporated into the PEDOT:PSS HTLs of BHJ-OSCs (Kadem et al., 2018), as illustrated in Figure 8A. The formation of SWCNTs:P3HT hybrids caused the conjugated polymer to twist around SWCNTs, resulting in nanoscale interconnected networks that facilitated the efficient transfer of holes between P3HT and SWCNTs, while selectively blocking the way for electron transport. This increased the shunt resistance and suppressed charge carrier recombination, resulting in a relatively higher electrical conductivity of 165 mS cm^−1^ for the acid-treated SWCNTs:P3HT hybrid when compared with 20 mS cm^−1^ for pristine P3HT. Consequently, devices with the PEDOT:PSS/acid-treated SWCNTs:P3HT HTL exhibited higher J_sc_; hence, a higher PCE of 2.52%, which outperformed devices based on P3HT/PEDOT:PSS HTLs that had a PCE of 2.48%. A significant increase in hole transport efficiency and electrical conductivity has also been observed after incorporating unzipped SWCNTs (uSWCNTs) into PEDOT:PSS to form a composite HTL of BHJ-OSCs (Zhang W. et al., 2020). This, in turn, increased the J_sc_ and FF, resulting in devices with a higher PCE of 14.60% when compared with 5.93 and 13.72% for the pristine uSWCNTs and PEDOT:PSS devices, respectively. The relatively poor performance of devices with pristine uSWCNTs was mainly associated with the inhomogeneity and high surface roughness of uSWCNTs, resulting in poor hole transport and current leakage due to charge carrier traps and short-circuits.

Boronic acid-functionalized MWCNTs (bf-MWCNTs) have also been incorporated into PEDOT:PSS and used as a composite HTL in OSCs (Dang et al., 2018). This increased the work function from 5.02 eV for the pristine PEDOT:SS HTL to 5.39 eV for the bf-MWCNTs/PEDOT:PSS HTL as illustrated in Figure 8B, and hence reduced the energy barrier by providing good energy level matching with the HOMO level of the PCDTBT donor and work function of the ITO anode. This subsequently improved the extraction and transportation of holes from the active layer to the anode, which significantly increased the hole mobility and electrical conductivity. Furthermore, the bf-MWCNTs/PEDOT:PSS-based devices were less affected by leakage current challenges due to their high shunt resistance (621 Ω cm^2^) and low series resistance (6.26 Ω cm^2^), which suppressed charge carrier recombination and increased the photogenerated current. This, in turn, increased the FF and J_sc_, resulting in MWCNTs/PEDOT:PSS-based devices with an optimum PCE of 6.95%, which outperformed the pristine MWCNT and PEDOT:PSS-based devices that had PCEs of 6.33 and 5.42%, respectively.

**FIGURE 9:**
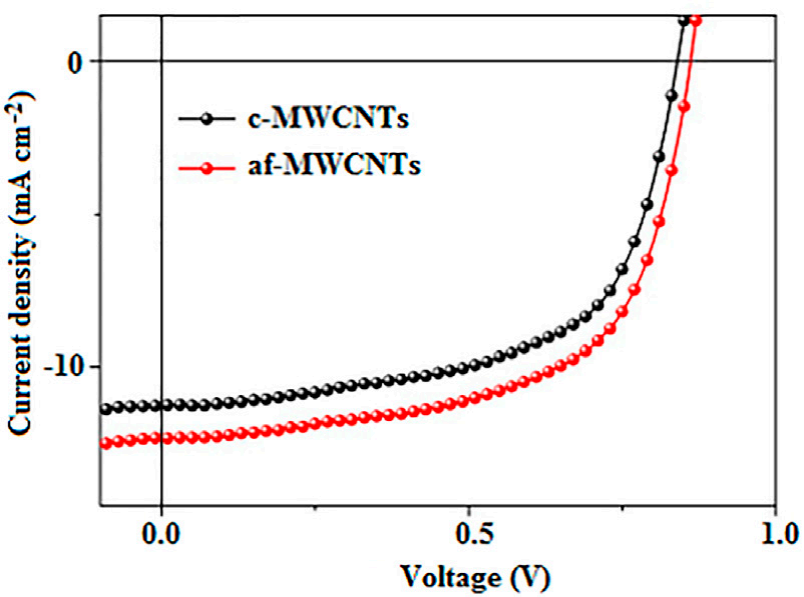
J-V characteristics of OSCs based on af-MWCNTs and c-MWCNTs. Adapted with permission (Zhang et al., 2019). Copyright 2019, Elsevier.

Also, the suitable work function of amino-functionalized MWCNTs (af-MWCNTs) of 5.22 eV enabled them to be used as HTLs in BHJ-OSCs to provide proper energy level alignment between the donor HOMO level (5.5 eV) and the work function of ITO (4.7 eV) (Zhang et al., 2019), which enhanced hole extraction and transport. In addition, the af-MWCNT-based devices exhibited a relatively lower series resistance (5.95 Ω cm^2^) and a higher shunt resistance (606.04 Ω cm^2^), when compared with 7.58 and 373.89 Ω cm^2^, respectively, for the control device based on carboxylated MWCNTs (c-MWCNTs). This indicated the presence of a smaller leakage current and lower charge carrier recombination rate in the OSCs with af-MWCNT HTLs, which improved their electrical conductivity, and hence increased their J_sc_ as illustrated in Figure 9, resulting in an optimum PCE of 6.97%, which outperformed the c-MWCNTs control device that had a PCE of 5.74%.

MWCNTs have also been integrated with poly (3-thiophene ethanol) (P3ThEt)-*graft*-PANI, and the resulting nanocomposites were used as HTLs in OSCs, which exhibited a relatively higher PCE of 5.30% when compared with 2.94 and 2.18% for the devices based on pristine MWCNTs and PEDOT:PSS, respectively (Sorkhishams et al., 2019). This was ascribed to the high optical transmittance (85–89%) of the MWCNTs/P3ThEt-*graft*-PANI HTL, which allowed more light to reach the active layer for effective exciton generation. In addition, the interpenetrated conductive networks formed by the MWCNTs/P3ThEt-*graft*-PANI nanocomposite facilitated the efficient transportation of photogenerated holes from the active layer to the anode. Also, the low R_rms_ of the smooth MWCNTs/P3ThEt-*graft*-PANI films resulted in a low series resistance and a high shunt resistance, which increased the electrical conductivity, reduced the leakage current and suppressed the recombination of charge carriers, thereby increasing the J_sc_ and FF, and subsequently increasing the device’s efficiency. Interestingly, the MWCNTs/P3ThEt-*graft*-PANI-based devices managed to retain above 75% of their initial PCE after storage for 30 days when compared with ∼23 and 60% for the pristine PEDOT:PSS and MWCNT-based devices, demonstrating their superior long-term stability.

Recently, the introduction of CNTs into PEDOT:PSS led to the formation of highly conducting interpenetrating networks throughout the HTL composite, which enhanced the transportation of holes, resulting in OSCs with relatively higher PCEs of 2.82 (Subramanyam et al., 2019), 3.76 (Subramanyam et al., 2020), and 6.44% (Oyeshola et al., 2020), when compared with the pristine PEDOT:PSS control devices. In addition, the chemical inertness of CNTs prevented the penetration of moisture and oxygen to the active layer, which significantly reduced the rapid degradation of materials. This enhanced the long-term stability of the CNTs/PEDOT:PSS-based devices when compared with the pristine PEDOT:PSS devices, which displayed a rapid drop in efficiency under the same environmental conditions due to the hygroscopic and acidic nature of PEDOT:PSS.

The photovoltaic parameters of OSCs with CNT-based HTLs, discussed in this work, are summarized in Table 3. Among these, the best PCE of 14.60% was obtained in more stable devices with uSWCNTs/PEDOT:PSS HTLs (Zhang W. et al., 2020). Hence, as a future research direction, the integration of CNTs with the conventional PEDOT:PSS HTL is envisaged to result in devices with excellent performance and stability, which is a significant step towards commercialization.

**TABLE 3:** Photovoltaic parameters of OSCs employing CNT-based HTLs.

HTL	V_oc_ (V)	J_sc_ (mA cm^−2^)	FF	PCE (%)	References
ZnO:SWCNTs/PEDOT:PSS	0.53	14.00	0.55	4.10	Mbuyise et al. (2017)
PEDOT:PSS/acid-treated SWCNTs:P3HT	0.55	8.48	0.54	2.52	Kadem et al. (2018)
PEDOT:PSS/SWCNTs:P3HT	0.53	8.70	0.55	2.52	Kadem et al. (2018)
uSWCNTs/PEDOT:PSS	0.85	23.39	0.73	14.60	Zhang W. et al. (2020)
uSWCNTs	0.51	21.50	0.54	5.93	Zhang W. et al. (2020)
MWCNTs	0.87	11.73	0.62	6.33	Dang et al. (2018)
bf-MWCNTs	0.88	12.51	0.63	6.95	Dang et al. (2018)
af-MWCNTs	0.87	12.65	0.64	6.97	Zhang et al. (2019)
c-MWCNTs	0.84	11.23	0.61	5.74	Zhang et al. (2019)
MWCNTs/P3ThEt-*graft*-PANI	0.68	12.85	0.61	5.30	Sorkhishams et al. (2019)
MWCNTs	0.63	9.42	0.50	2.94	Sorkhishams et al. (2019)
CNTs/PEDOT:PSS	0.65	9.86	0.44	2.82	Subramanyam et al. (2019)
CNTs/PEDOT:PSS	0.66	10.83	0.53	3.76	Subramanyam et al. (2020)
CNTs/PEDOT:PSS	0.72	11.02	0.72	6.44	Oyeshola et al. (2020)

### Electron Transport Layer

ETLs are low work function n-type materials that reduce the energy barrier between the LUMO level of the electron acceptor and work function of the cathode; hence, they provide an Ohmic contact at the interface between the active layer and cathode (Amusan et al., 2019). This improves the extraction and transportation of electrons from the photoactive layer to the cathode, while selectively blocking hole transport, thereby suppressing electron and hole recombination (Soh et al., 2019).

An assortment of ETL materials, including low work function metals or related salts, e.g., Ca (Anagnostou et al., 2019; Ghosekar and Patil, 2019; Lian et al., 2019; Song et al., 2019; Yu et al., 2019) and LiF (Zhao and Alford, 2018; Lee et al., 2019; Zheng et al., 2019; Adedeji et al., 2020; Li et al., 2020b), and n-type semiconducting metal oxides, e.g., ZnO (Upama et al., 2017; Ahmad et al., 2019; Frankenstein et al., 2019; Zhang X. et al., 2020; Usmani et al., 2021) and TiO_2_ (Lin et al., 2013; Sun et al., 2016; Al-hashimi et al., 2018; Abdallaoui et al., 2020; Chaudhary et al., 2021), have been commonly used to fabricate OSCs. However, Ca and LiF are usually deposited by thermal evaporation in a high vacuum environment at high temperature, which is expensive, complicated and incompatible with flexible devices; hence, making them unfavourable. Although, ZnO and TiO_2_ have merits, such as low-cost, solution processability, non-toxicity, facile availability and high optical transmittance in the visible region (Mutlu et al., 2019; Wei et al., 2019), their choice is limited by poor charge transport due to defects incurred during film growth, which increases charge carrier recombination (Mohamad Noh et al., 2018), thereby reducing the efficiency of devices. This challenge can be addressed by high-temperature annealing to improve the physicochemical properties of ZnO and TiO_2_ films, but high-temperature annealing is not compatible with flexible devices (Wang et al., 2018). As a consequence, carbon-based materials, particularly CNTs, have recently attracted considerable research interest as potential alternatives for ETL applications owing to their low-cost, solution-processability, high optical transparency, good electrical conductivity, high flexibility and superior stability.

**FIGURE 10:**
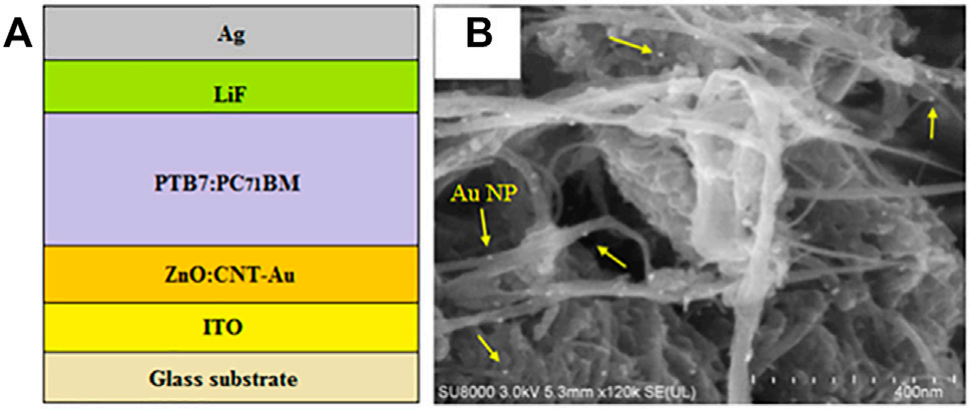
**(A)** Schematic diagram of a conventional BHJ-OSC with a ZnO:CNTs-Au composite ETL and **(B)** SEM micrograph of the CNTs-Au film. Adapted with permission (Hu et al., 2016). Copyright 2016, Elsevier.

In this regard, CNT-Au nanocomposites have been incorporated into ZnO NPs and employed as hybrid ETLs in OSCs (Hu et al., 2016; Li et al., 2019), as illustrated in Figure 10A. The CNTs provided a suitable template for the *in situ* growth of ZnO NPs, resulting in uniform films with low defect density and good electrical conductivity, whereas the Au NPs, bound uniformly to the CNTs as illustrated in Figure 10B, inducing a surface plasmon effect, which enhanced the absorption of light in the active layer. This improved the generation of excitons, suppressed the recombination of charge carriers and facilitated the smooth transportation of electrons to the cathode. In addition, the incorporation of CNTs-Au into ZnO reduced the work function of the composite ETL, which provided better energy level alignment between the active layer and cathode for the effective extraction of electrons from the active layer and transportation to the cathode. This, in turn, increased exciton generation and electron mobility, as well as reducing charge carrier recombination, thereby increasing the J_sc_ and FF. Consequently, the ZnO:CNTs-Au-based devices displayed relatively higher PCEs of 7.90 (Hu et al., 2016) and 10.49% (Li et al., 2019), respectively, when compared with 7.00 and 8.41% for the corresponding pristine ZnO reference devices.

**FIGURE 11:**
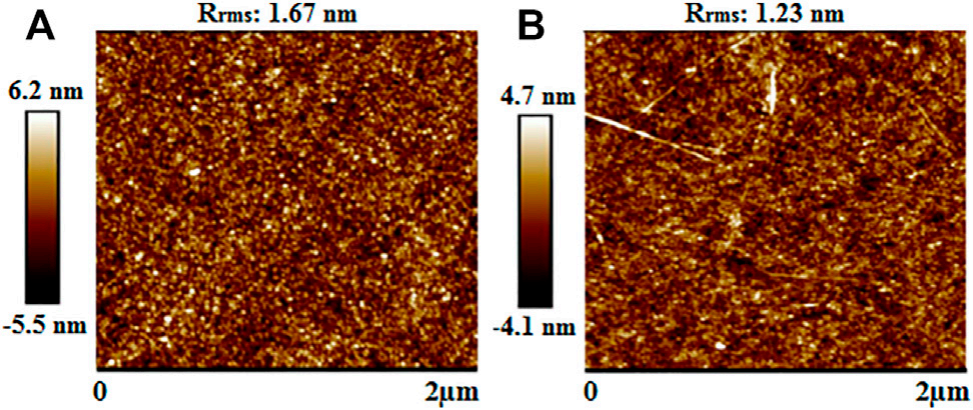
AFM micrographs of **(A)** pristine ZnO NPs and **(B)** ASP-SWCNTs on ZnO NPs. Adapted with permission (Lee et al., 2020). Copyright 2020, American Chemical Society.

Recently, alcohol-soluble polyfluorene (ASP)-wrapped SWCNTs have been incorporated into ZnO NPs and employed as composite ETLs in OSCs (Lee et al., 2020). ASP facilitated the individual dispersion of SWCNTs in solution, which resulted in smooth films with low R_rms_ as shown in Figure 11, thereby addressing the issue of leakage current due to short-circuits, originating from the agglomeration and bundling of poorly dispersed SWCNTs in rough films. In addition, the ASP-SWCNTs played a significant role in increasing the internal quantum efficiency, balancing the mobility between holes and electrons, and reducing charge carrier recombination, which increased the J_sc_ and FF. This resulted in PCEs of 9.45 and 14.37% for the devices using the poly([2,6′-4,8-di(5-ethylhexylthienyl)benzo[1,2-b;3,3-b]dithiophene] (Subramanyam et al., 2020)) (PTB7-Th):PC_71_BM and poly[(2,6-(4,8-bis(5-(2-ethylhexyl-3-fluoro)thiophen-2-yl)-benzo [1,2-b:4,5-b']dithiophene))-alt-(5,5-(1′,3′-di-2-thienyl-5′,7′-bis(2-ethylhexyl)benzo [1′,2′-c:4′,5′-c']dithiophene-4,8-dione)]: (2,20-((2Z, 20Z)-((12,13-bis(2-ethylhexyl)-3,9-diundecyl-12,13-dihydro-[1,2,5]thiadiazolo [3,4-e]thieno [2,"30':4′,50]thieno[20,30:4,5]pyrrolo[3,2-g]thieno[20,30:4,5]thieno[3,2-b]indole-2,10-diyl)bis(methanylylidene))bis(5,6-difluoro-3-oxo-2,3-dihydro-1H-indene-2,1-diylidene))dimalononitrile) (PM6:Y6) active layer blends, respectively, which outperformed the corresponding pristine ZnO devices that had PCEs of 7.97 and 13.73%.

The photovoltaic parameters of OSCs with CNT-based ETLs, discussed in this study, are summarized in Table 4. From these, the highest PCE of 14.37% was achieved by OSCs with the ZnO:ASP-SWCNTs ETL (Lee et al., 2020), demonstrating the suitability of CNT-based ETLs in the fabrication of highly efficient devices.

**TABLE 4:** Photovoltaic parameters of OSCs employing CNT-based ETLs.

ETL	V_oc_ (V)	J_sc_ (mA cm^−2^)	FF	PCE (%)	References
ZnO:CNTs	0.72	16.37	0.64	7.60	Hu et al. (2016)
ZnO:CNTs-Au	0.72	16.81	0.65	7.90	Hu et al. (2016)
ZnO:CNTs-Au	0.80	18.37	0.72	10.49	Li et al. (2019)
ZnO:ASP-SWCNTs	0.80	17.72	0.66	9.45	Lee et al. (2020)
ZnO:ASP-SWCNTs	0.87	24.88	0.66	14.37	Lee et al. (2020)

## Conclusion and Outlook

This review has presented the recent trends in the application of CNTs in electrodes, active layers and charge transport layers of OSCs, in response to the increasing demand to develop alternative materials that can replace or modify the traditional materials, which are currently producing devices with relatively low PCE and poor long-term stability. Among the possible alternatives, carbon-based materials, especially CNTs, are more appealing due to their solution processability, non-toxicity, the natural abundance of carbon, low-cost, competitive optoelectronic properties and excellent stability. However, the efficiency of CNT-based OSCs is still relatively lower than that of the state-of-the-art OSCs fabricated with traditional materials due to drawbacks, such as the relatively low visible region optical transparency of CNT-based electrodes and charge transport layers, which limit the passage of incoming photons to the active layer, thereby reducing the exciton generation rate, and hence lowering the photogeneration of current. In addition, the sheet resistance of CNT-based electrodes is relatively higher than that of commonly used ITO electrodes. This limits the efficient collection and transfer of photogenerated current from the charge transport layers to the external circuit, thereby impairing device performance. Nonetheless, the tunable band gap of CNTs, in addition to selective charge carrier transportation and blocking capabilities of CNT-based charge transport layers, enable the provision of an Ohmic contact at the photoactive layer-electrode interface, thereby improving charge carrier extraction and mobility, which increases the efficiency of devices. Also, the hydrophobic nature of CNTs helps to prevent the diffusion of moisture and air into the photoactive layer, which protects the photoactive layer materials from degradation, thereby enhancing the stability of OSCs. Furthermore, the large specific surface area of CNTs provides a large interfacial area between the donor and acceptor, which enhances exciton dissociation, in addition to the highly conductive CNT interpenetrating network, which improves the transfer of electrons and holes to their respective electrodes before recombination, thereby improving device performance. Therefore, as a future research direction, optimization of the optoelectronic properties of CNTs via approaches, such as chemical doping and formation of nanocomposites, is envisaged to pave the way for the commercialization of CNT-based OSCs through the fabrication of highly efficient, sustainable and cost-effective devices.
